# Production of 9,21-dihydroxy-20-methyl-pregna-4-en-3-one from phytosterols in *Mycobacterium neoaurum* by modifying multiple genes and improving the intracellular environment

**DOI:** 10.1186/s12934-021-01717-w

**Published:** 2021-12-23

**Authors:** Chen-Yang Yuan, Zhi-Guo Ma, Jing-Xian Zhang, Xiang-Cen Liu, Gui-Lin Du, Jun-Song Sun, Ji-Ping Shi, Bao-Guo Zhang

**Affiliations:** 1 Lab of Biorefinery, Shanghai Advanced Research Institute, Chinese Academy of Sciences, No. 99 Haike Road, Pudong, Shanghai, 201210 China; 2 School of Life Science and Technology, ShanghaiTech University, Shanghai, 201210 China; 3 University of Chinese Academy of Sciences, Beijing, 100049 China

**Keywords:** 9,21-dihydroxy-20-methyl-pregna-4-en-3-one (9-OH-4-HP), *kstD*, *hsd4A*, *fadA5*, Intracellular environment

## Abstract

**Background:**

Steroid drugs are essential for disease prevention and clinical treatment. However, due to intricated steroid structure, traditional chemical methods are rarely implemented into the whole synthetic process for generating steroid intermediates. Novel steroid drug precursors and their ideal bacterial strains for industrial production have yet to be developed. Among these, 9,21-dihydroxy-20-methyl-pregna-4-en-3-one (9-OH-4-HP) is a novel steroid drug precursor, suitable for the synthesis of corticosteroids. In this study, a combined strategy of blocking Δ^1^-dehydrogenation and the C19 pathway as well as improving the intracellular environment was investigated to construct an effective 9-OH-4-HP-producing strain.

**Results:**

The Δ^1^-dehydrogenation-deficient strain of wild-type *Mycobacterium neoaurum* DSM 44074 produces 9-OH-4-HP with a molar yield of 4.8%. *Hsd4A*, encoding a β-hydroxyacyl-CoA dehydrogenase, and *fadA5*, encoding an acyl-CoA thiolase, were separately knocked out to block the C19 pathway in the Δ^1^-dehydrogenation-deficient strain. The two engineered strains were able to accumulate 0.59 g L^−1^ and 0.47 g L^−1^ 9-OH-4-HP from 1 g L^−1^ phytosterols, respectively. Furthermore, *hsd4A* and *fadA5* were knocked out simultaneously in the Δ^1^-dehydrogenation-deficient strain. The 9-OH-4-HP production from the Hsd4A and FadA5 deficient strain was 11.9% higher than that of the Hsd4A deficient strain and 40.4% higher than that of the strain with FadA5 deficiency strain, respectively. The purity of 9-OH-4-HP obtained from the Hsd4A and FadA5 deficient strain has reached 94.9%. Subsequently, the catalase *katE* from *Mycobacterium neoaurum* and an NADH oxidase, *nox*, from *Bacillus subtilis* were overexpressed to improve the intracellular environment, leading to a higher 9-OH-4-HP production. Ultimately, 9-OH-4-HP production reached 3.58 g L^−1^ from 5 g L^−1^ phytosterols, and the purity of 9-OH-4-HP improved to 97%. The final 9-OH-4-HP production strain showed the best molar yield of 85.5%, compared with the previous reported strain with 30% molar yield of 9-OH-4-HP.

**Conclusion:**

KstD, Hsd4A, and FadA5 are key enzymes for phytosterol side-chain degradation in the C19 pathway. Double deletion of *hsd4A* and *fadA5* contributes to the blockage of the C19 pathway. Improving the intracellular environment of *Mycobacterium neoaurum* during phytosterol bioconversion could accelerate the conversion process and enhance the productivity of target sterol derivatives.

**Supplementary Information:**

The online version contains supplementary material available at 10.1186/s12934-021-01717-w.

## Background

Steroid drugs, including mineralocorticoids, glucocorticoids, and sex hormones, are crucial in the prevention and clinical treatment of various diseases [1, 2]. In industrial manufacturing, two major valuable intermediates of sterols, C19 steroids, and C22 steroids, can be used to synthesize sex and adrenocortical hormones. However, traditional chemical methods are rarely implemented in the whole synthetic processes of modifying steroid intermediates due to the intricate steroid structure. Thus, the pursuit of novel steroid drug precursors has intrigued some researchers. Certain C22 steroids are ideal precursors for steroid drug synthesis [3]. Among these steroids, 9,21-dihydroxy-20-methyl-pregna-4-en-3-one (9-OH-4-HP) is a valuable and novel steroid derivative for the synthesis of corticosteroids because of its substituents at positions C-9 and C-21. 9-OH-4-HP was commonly identified as a by-product during bioconversion of sterol to 9-hydroxy steroid derivatives in several *Mycobacterium* species, such as *Mycobacterium* sp. 2–4M that produces a 1.5–1.6% molar yield of 9-OH-4-HP [4]. However, ideal industrial strains for 9-OH-4-HP production have not yet to be developed.

Due to its mild reaction conditions in the process of steroid synthesis, the microbial transformation has caught increasing attention for medicinal chemists [5]. Among these reactions, Δ^1^-dehydrogenation was one of the most thoroughly investigated the Δ^1^-dehydrogenation of the sterol skeleton was catalyzed by 3-ketosteroid-1(2)-dehydrogenase (KstD) [6]. Strains with inactivation of KstD generally leads to various 9α-hydroxy derivatives after culture with steroids, such as 9-hydroxy-androst-4-ene-3,17-dione (9-OH-AD), and 9-OH-4-HP. 9α-hydroxy derivatives are important precursors in the manufacture of several modern glucocorticoid drugs with a halogen at the 9α position [7]. Other than industrial strains with KstD deficiency that accumulate 9α-hydroxy derivatives after culture with sterol [6], a few wild-type strains of *Mycobacterium* have been reported to be able to produce 9-OH-AD [4].

**Fig. 1:**
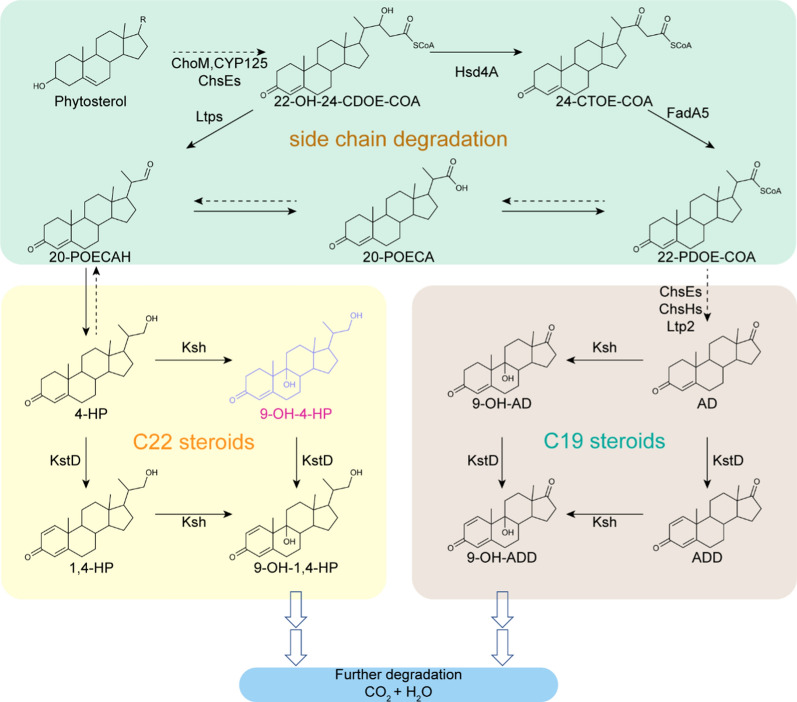
A schematic diagram of physterols side-chain degradation in *Mycobacterium*. ChoM, Cholesterol oxidase; CYP125, cytochrome P450 125; ChsEs, acyl-CoA dehydrogenases; ChsHs, 3-oxo-23,24-bisnorchol-4,17(20)-dien-22-oyl-CoA-hydratase; Ltp2, lipid transfer protein 2; Hsd4A, 17β-hydroxysteroid dehydrogenase/β-hydroxyacyl-CoA dehydrogenase; FadA5, acetyl-CoA acetyltransferase/thiolase; KstD, 3-ketosteroid-Δ^1^-dehydrogenase; KSH, 3-ketosteroid-9α-hydroxylase; 22-OH-24-CDOE-CoA, 22-hydroxy-3,24-dioxo-4-ene-cholest-CoA; 24-CTOE-CoA, 3,22,24-trioxo-4-ene-cholest-CoA; 22-PDOE-CoA, 3,22-dioxo-4-ene-pregna-CoA; 20-POECA, 3-oxo-4-ene-pregna-20-carboxylic acid; 20-POECAH, 3-oxo-4-ene-pregna-20-carboxyaldehyde

Dual competing pathways, the overwhelming C19 steroid pathway, and the C22 steroid pathway are involved into phytosterol side-chain degradation (Fig. 1). Recently, the 17-hydroxysteroid/22-OH-BNC-CoA dehydrogenase Hsd4A was found relevant to C22 steroid formation [3]. Inactivation of Hsd4A enabled the production of C22 steroids from sterols. For example, *M. neoaurum* NwIB-XII accumulates two C19 steroids as the main products after culture with cholesterol, androst-4-ene-3,17-dione (AD) and androst-1,4-diene-3,17-dione (ADD). While the *hsd4A* knockout strain of *M. neoaurum* NwIB-XII accumulates 21-hydroxy-20-methyl-pregna-4-en-3-one (4-HP) and 21-hydroxy-20-methyl-pregna-1,4-dien-3-one (1,4-HP) as the main products after culture with cholesterol, which both are C22 steroids. Nevertheless, C19 steroids still accumulated in the *hsd4A* knockout strain after culture with sterols, indicating an incomplete blockage of the C19 steroid pathway [3]. FadA5, a thiolase, which lies in the downstream of Hsd4A, catalyzes the thiolysis of 3,22,24-trioxo-4-ene-cholest-CoA (24-CTOE-CoA) to 3,22-dioxo-4-ene-pregna-CoA (22-PDOE-CoA) (Fig. 1) [8]. A FadA5-deficient strain of *M. neoaurum* NwIB-XII also accumulates both 4-HP and 1,4-HP as the main products, indicating that the deletion of *fadA5* may contribute to a further blockage of the C19 pathway. Therefore, *hsd4A and fadA5* are important targets for modification by genetic engineering to develop microorganisms that can transform sterols into the valuable steroidal intermediate 9-OH-4-HP.

Phytosterols are reported to inhibit the cell growth by reducing the utilization of carbon sources, which hinder the steroids biosynthesis progress with phytosterols as a substrate [9]. A steady-state intracellular environment could be beneficial for phytosterol degradation by *Mycobacterium*. Toxic steroid intermediates cause cells to produce reactive oxygen species (ROS), including hydrogen peroxide (H_2_O_2_), during aerobic metabolism. A high level of H_2_O_2_ might harm cell growth, hence slowing the rate of phytosterol degradation and decreasing the yield of metabolites [10], and vice versa. In addition, during the phytosterol degradation process, intracellular nicotinamide adenine dinucleotides (NAD^+^ and NADH) are consumed, which participate in the multistep reactions such as dehydrogenation. NAD^+^/NADH regeneration and maintenance of the redox balance are considered the rate-limiting factors in the steroid degradation pathway [10, 11]. Manipulation of NAD^+^/NADH contents could enhance the production of AD and ADD to various degrees [10–12]. Overexpression of NADH oxidase in *M. neoaurum* JC-12 increased ADD production by 43% [10]. Thus, the elimination of H_2_O_2_ and regeneration of NAD^+^ could contribute to higher concentrations of phytosterol metabolites.

Herein, an engineered strain of *M. neoaurum* DSM 44,074, as a sterol consumer with no steroid final products, was constructed for the bioconversion of phytosterols to 9-OH-4-HP. A *kstD* knockout strain was constructed based on *M. neoaurum* DSM 44074, and the C19 steroid pathway was further blocked by knocking out both *hsd4A* and *fadA5*. By improving the intracellular environment, an efficient 9-OH-4-HP-producing strain was generated. This strain may contribute to the development of steroid drug precursors.

## Results

### Accumulation of 9α-hydroxy derivatives

**Fig. 2:**
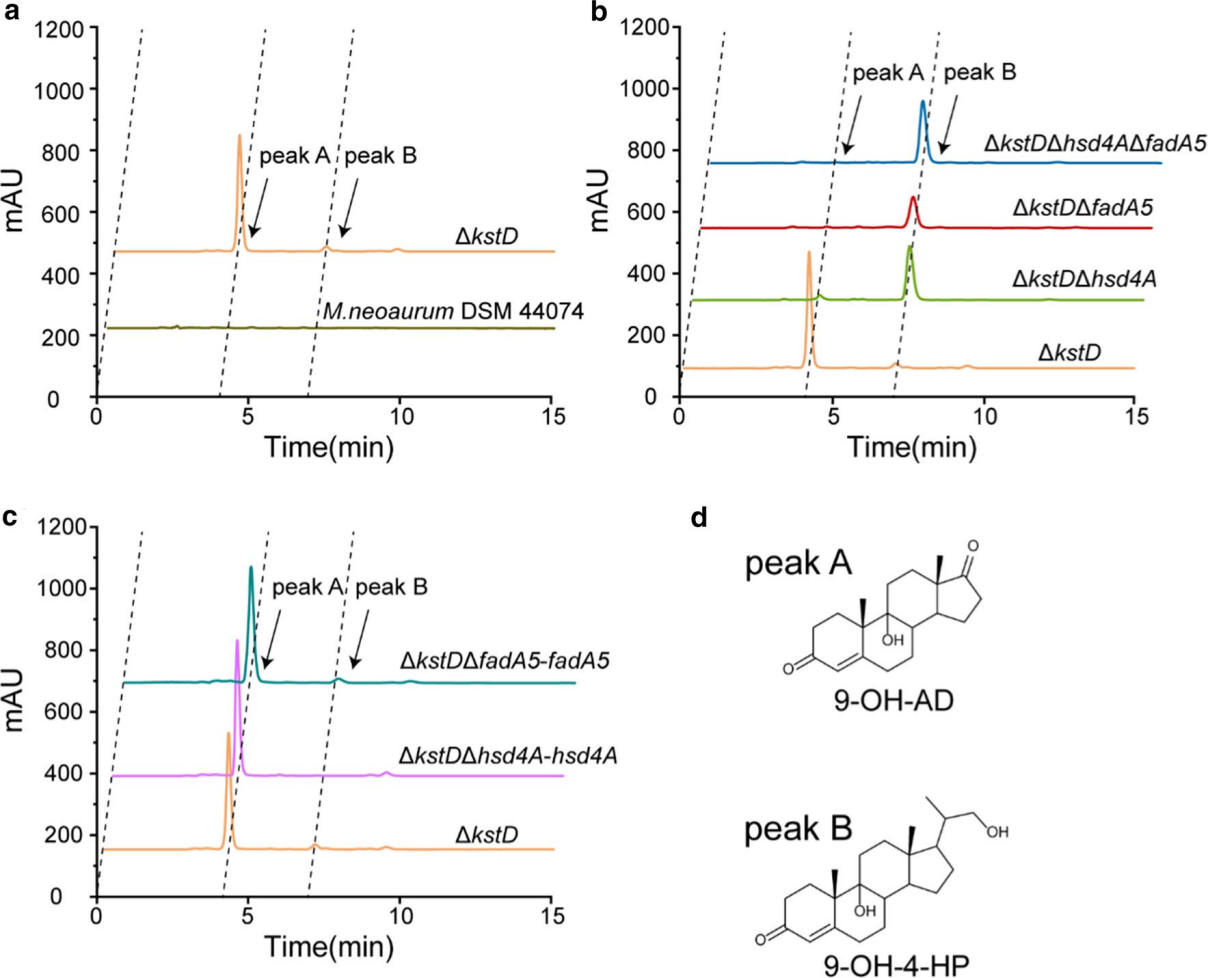
Phenotypic analyses of the metabolites of phytosterol by *M. neoaurum* DSM 44074 and its derivative strains. **a** HPLC chromatogram comparison of the products of *M. neoarum* DSM 44704 and Δ*kstD* with 1 g L^−1^ phytosterols feed. **b** HPLC chromatogram comparison of the products of Δ*kstD*, Δ*kstD*Δ*hsd4A*, Δ*kstD*Δ*fadA5* and Δ*kstD*Δ*hsd4A*Δ*fadA5* with 1 g L^−1^ phytosterols feed. **c** HPLC chromatogram comparison of the products of Δ*kstD*, *hsd4A* complement strain Δ*kstD*Δ*hsd4A*-*hsd4A* and *fadA5* complement strain Δ*kstD*Δ*fadA5*-*fadA5*, with 1 g L^−1^ phytosterols feed. **d** Structure of peak A, 9-OH-AD, and peak B, 9-OH-4-HP

**Fig. 3:**
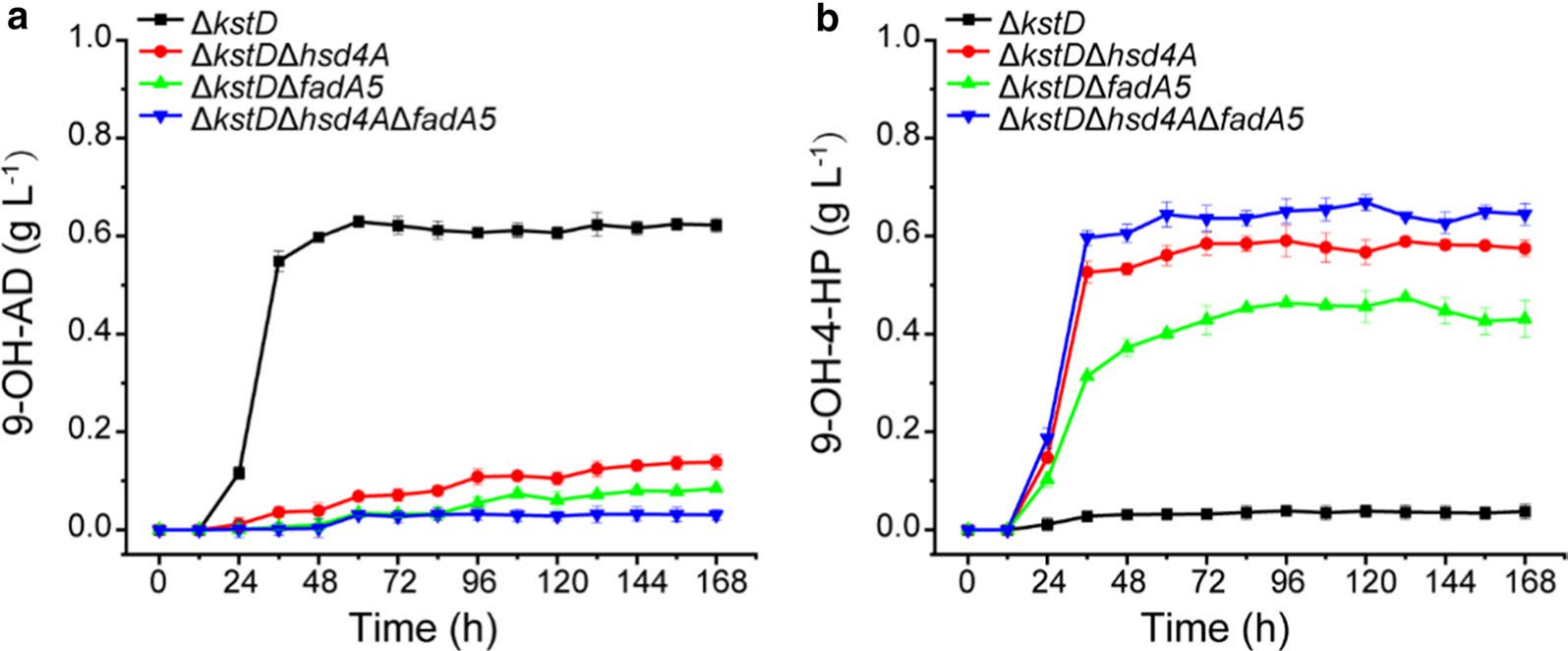
9-hydroxy steroids accumulation from 1 g L^−1^ phytosterols. **a** Time course of 9-OH-AD accumulation; **b** time course of 9-OH-4-HP accumulation; single deletion of *hsd4A* or *fadA5* caused increment of 9-OH-4-HP, and double deletion of *hsd4A* and *fadA5* could obviously increase the productivity and purity of 9-OH-4-HP

To eliminate Δ^1^-dehydrogenation and accumulate 9α-hydroxy derivatives from phytosterols (Fig. 1), *kstDs* were beforehand identified and subsequently knocked out from the genome of the wild-type strain *M. neoaurum* DSM 44074, a steroid-degrading *Mycobacterium* that can completely degrade phytosterols into CO_2_ and H_2_O [13]. The genome of *M. neoaurum* DSM 44074 was sequenced as described in the Methods section. Three putative *kstD* genes (gene 5102 for *kstD1*, gene 5236 for *kstD2*, and gene 5233 for *kstD3*) were identified in *M. neoaurum* DSM 44704. *kstDs* was successfully knocked out from the genome of *M. neoaurum* DSM 44704 with a CRISPR-assisted nonhomologous end-joining strategy as described in the Methods section, resulting in a mutant strain Δ*KstD*. The cell growth of the *ΔkstD* strain showed no significant difference from that of the wild-type strain (Additional file 1: Fig. S1). The wild-type *M. neoaurum* DSM 44074 strain and the genetically modified strain Δ*kstD* were incubated with phytosterols for 168 h. Compared with the wild-type strain *M. neoaurum* DSM 44074, which showed no detectable product by HPLC analysis (Fig. 2a), the Δ*kstD* strain produced 9-OH-AD as the main product with a retention time of 4.2 min (Fig. 2a, peak A), along with 9-OH-4-HP as a by-product with a retention time of 7.1 min (Fig. 2a, peak B). No ADD was detected during phytosterol bioconversion by Δ*kstD*, proving the elimination of Δ^1^-dehydrogenation by *kstDs* knockout. When MP01 medium plus 1 g L^−1^ phytosterols was used for the incubation of the Δ*kstD* strain, 0.62 g L^−1^ 9-OH-AD and 0.04 g L^−1^ 9-OH-4-HP were produced within 60 h (Fig. 3a and b). The molar yield of 9-OH-AD reached 84.9%. 9-OH-4-HP was the by-product during phytosterol bioconversion by Δ*kstD*, with a molar yield of 4.8%, and 5.6% purity (Table 1). 9α-hydroxy derivatives successfully accumulated during phytosterol bioconversion by engineered *Mycobacterium*, but the purity and yield of 9-OH-4-HP didn’t show satisfactory.

**Table 1:** Relative production purity of *M. neoaurum* DSM 44074 and its derivative strains

Strain	Relative purity (%)			
	9-OH-AD	9-OH-4-HP	AD	Others
DSM 44704	0	0	0	0
Δ*kstD*	90.4 ± 3.1	5.6 ± 2.5	1.9 ± 0.2	2.1 ± 0.8
Δ*kstD*Δ*hsd4A*	7.2 ± 2.2	88.6 ± 1.3	2.4 ± 0.5	1.8 ± 1.3
Δ*kstD*Δ*fadA5*	8.0 ± 1.9	86.0 ± 3.5	3.3 ± 0.4	2.7 ± 0.5
Δ*kstD*Δ*hsd4A*-*hsd4A*	90.0 ± 2.2	5.2 ± 1.7	1.8 ± 0.2	3.0 ± 0.3
Δ*kstD*Δ*fadA5*-*fadA5*	88.5 ± 3.6	4.4 ± 2.3	1.3 ± 0.1	5.8 ± 1.2
Δ*kstD*Δ*hsd4A*Δ*fadA5*	2.0 ± 1.1	94.9 ± 1.2	0.3 ± 0.1	2.8 ± 0.3
Δ*kstD*Δ*hsd4A*Δ*fadA5*-NK	2.0 ± 1.1	97.0 ± 1.1	0.3 ± 0.1	0.7 ± 0.3

### Construction of a 9-OH-4-HP-producing strain

Dual pathways, the C19 steroid pathway, and the C22 steroid pathway compete during phytosterol side-chain degradation (Fig. 1). The C19 steroid pathway is considered as the dominant pathway in *M. neoaurum* DSM 44074 because Δ*kstD* produces 9-OH-AD as the main product along with little 9-OH-4-HP as a by-product after culture with phytosterols. The two pathways diverge at 22-hydroxy-3,24-dioxo-4-ene-cholest-CoA (22-OH-24-CDOE-CoA), which could be Δ^22^-dehydrogenated by the β-hydroxyacyl-CoA dehydrogenase Hsd4A and generate 3,22,24-trioxo-4-ene-cholest-CoA (24-CTOE-CoA). 24-CTOE-CoA could subsequently be catalyzed by the thiolase FadA5, leading the phytosterol degradation flux to the C19 pathway (Fig. 1).

Thus, to construct a 9-OH-4-HP-producing strain, *hsd4A* and *fadA5* were identified in the genome of *M. neoaurum* DSM 44074 and separately knocked out in Δ*kstD*, resulting in the strains Δ*kstD*Δ*hsd4A* and Δ*kstD*Δ*fadA5*.

The cell growth of Δ*kstD*Δ*hsd4A* and Δ*kstD*Δ*fadA5* showed no significant difference from that of the wild-type strain *M. neoaurum* DSM 44074 (Additional file 1: Fig. S1). The strains Δ*kstD*Δ*hsd4A* and Δ*kstD*Δ*fadA5* were cultured with phytosterols for 168 h, and the metabolites were analyzed by HPLC (Fig. 2b). As shown in Fig. 3b, 9-OH-4-HP, successfully accumulated in both strains Δ*kstD*Δ*hsd4A* and Δ*kstD*Δ*fadA5* as a major production. The purity of 9-OH-4-HP from the Δ*kstD*Δ*hsd4A* and Δ*kstD*Δ*fadA5* strains was 88.6% and 86.0%, respectively. Still, both strains showed a small amount of 9-OH-AD accumulation (Fig. 3a). The purity of 9-OH-AD from strains Δ*kstD*Δ*hsd4A* and Δ*kstD*Δ*fadA5* were 7.2% and 8.0%, respectively. After culture with 1 g L^−1^ phytosterols in MP01 medium, the strain Δ*kstD*Δ*hsd4A* accumulated 0.59 g L^−1^ 9-OH-4-HP and 0.13 g L^−1^ 9-OH-AD, while 0.47 g L^−1^ 9-OH-4-HP and 0.08 g L^−1^ 9-OH-AD were obtained from strain Δ*kstD*Δ*fadA5*. Interestingly, the molar yields of 9-OH-4-HP and 9-OH-AD from strain Δ*kstD*Δ*fadA5* were both lower than that of from strain Δ*kstD*Δ*hsd4A*. The molar yield of 9-OH-4-HP from strain Δ*kstD*Δ*fadA5* was 20.3% lower than that from strain Δ*kstD*Δ*hsd4A*, and the molar yield of 9-OH-AD from strain Δ*kstD*Δ*fadA5* which was 38.5% lower than that from strain Δ*kstD*Δ*hsd4A*.

Considering that 9-OH-AD still accumulated in both the Δ*kstD*Δ*hsd4A* and Δ*kstD*Δ*fadA5* strains, the C19 steroid pathway of the phytosterol degradation pathway was not probably completely blocked in either strain. To enhance the purity and production of 9-OH-4-HP and obstruct the yield of 9-OH-AD, *hsd4A and fadA5* were simultaneously knocked out in strain Δ*kstD*, resulting in the strain *ΔkstDΔhsd4AΔfadA5*. The cell growth of the *ΔkstDΔhsd4AΔfadA5* strain showed a similar trend with the wild-type strain *M. neoaurum* DSM 44,074 (Fig. S1). As shown in Fig. 2b, after culture with phytosterols, the strain Δ*kstD*Δ*hsd4A*Δ*fadA5* accumulated 9-OH-4-HP as the main product, while the accumulation of 9-OH-AD was significantly decreased, compared with the strains Δ*kstD*Δ*hsd4A* and Δ*kstD*Δ*fadA5*. The purity of 9-OH-4-HP from strain Δ*kstD*Δ*hsd4A*Δ*fadA5* was 94.9%, obviously higher than those from strains Δ*kstD*Δ*hsd4A* and Δ*kstD*Δ*fadA5*. Meanwhile, the accumulation of 9-OH-AD from strain Δ*kstD*Δ*hsd4A*Δ*fadA5* was significantly decreased at a purity of 2.0%. After culture with 1 g L^−1^ phytosterols, 0.66 g L^−1^ 9-OH-4-HP was obtained from strain Δ*kstD*Δ*hsd4A*Δ*fadA5*, which is 11.9% more than that from strain Δ*kstD*Δ*hsd4A* and 40.4% more than that from strain Δ*kstD*Δ*fadA5* (Fig. 3b). The purity and production of 9-OH-4-HP from strain Δ*kstD*Δ*hsd4A*Δ*fadA5* were both higher than those from strains Δ*kstD*Δ*hsd4A* and Δ*kstD*Δ*fadA5*, indicating that the double knockout of *hsd4A* and *fadA5* could effectively block the accumulation of AD homologues.

To verify the functions of *hsd4A* and *fadA5* during phytosterol degradation, Δ*kstD*Δ*hsd4A*-*hsd4A*, the *hsd4A* complementation strain of strain Δ*kstD*Δ*hsd4A*, and Δ*kstD*Δ*fadA5*-*fadA5*, the *fadA5* complementation strain of Δ*kstD*Δ*fadA5* were also constructed. As shown in Fig. 2c, the accumulation of 9-OH-AD was recovered when the two complementation strains were cultured with phytosterols. The purities of 9-OH-AD from Δ*kstD*Δ*hsd4A*-*hsd4A* and Δ*kstD*Δ*fadA5*-*fadA5* were 90.0% and 88.5%, respectively, which are consistent with those of strain Δ*kstD*. These results indicate that Hsd4A and FadA5 are key enzymes in the C19 steroid pathway during phytosterol side-chain degradation. Phylogenetic trees of Hsd4A and FadA5 were constructed to elucidate the evolutionary relationship of the two enzymes (Additional file 1: Fig. S2).

### Evaluation of the 9-OH-4-HP producer

After culture with 1 g L^−1^ phytosterols, the molar yield of 9-OH-4-HP from Δ*kstD*Δ*hsd4A*Δ*fadA5* was 78.9%. To evaluate the ability of Δ*kstD*Δ*hsd4A*Δ*fadA5* of transforming phytosterols into 9-OH-4-HP, higher concentrations of phytosterols were incubated with Δ*kstD*Δ*hsd4A*Δ*fadA5*.

**Fig. 4:**
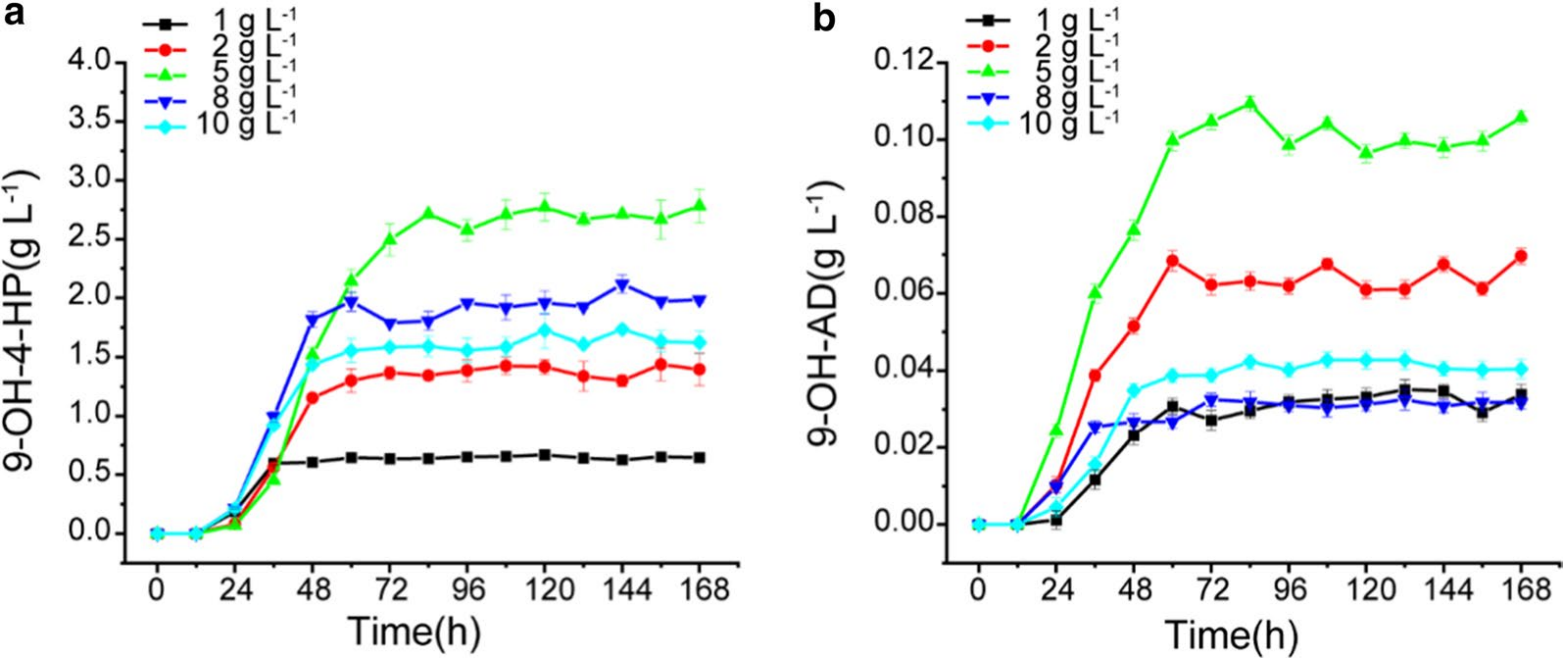
9-hydroxy steroids accumulation of Δ*kstD*Δ*hsd4A*Δ*fadA5* from different concentrations of phytosterols. **a** Time course of 9-OH-4-HP accumulation; **b** time course of 9-OH-AD accumulation; As the phytosterols concentration increased, the ability of the strain Δ*kstD*Δ*hsd4A*Δ*fadA5* to transform phytosterols was inhibited

As shown in Fig. 4a, the yields of 9-OH-4-HP from the bioconversion of 2 g L^−1^, 5 g L^−1^, 8 g L^−1^, and 10 g L^−1^ phytosterols by Δ*kstD*Δ*hsd4A*Δ*fadA5* were 1.43 g L^−1^, 2.78 g L^−1^, 1.98 g L^−1^, and 1.73 g L^−1^, respectively. 9-OH-AD was also obtained during the incubation, showing yields of 0.06 g L^−1^, 0.10 g L^−1^, 0.03 g L^−1^, and 0.04 g L^−1^, respectively (Fig. 4b). The molar yields of 9-OH-4-HP from different concentrations of phytosterols are listed in Table 2. The highest molar yield, 84.8% of 9-OH-4-HP, was obtained when strain Δ*kstD*Δ*hsd4A*Δ*fadA5* was cultured with 2 g L^−1^ phytosterols. Thence a downward trend in the molar yield of 9-OH-4-HP appeared as the concentration of phytosterols increased from 2 g L^−1^ to 10 g L^−1^. However, the purity of 9-OH-4-HP remained stable. Previous research has reported that phytosterols and their metabolites could be noxious to cells during bioconversion [9, 14–16], for the poor performance of Δ*kstD*Δ*hsd4A*Δ*fadA5* under high concentration phytosterols bioconversion.

**Table 2:** Maximum yield and molar yield of 9-OH-4-HP from Δ*kstD*Δ*hsd4A*Δ*fadA5* and Δ*kstD*Δ*hsd4A*Δ*fadA5*-NK

Phytosterols concentration (g L^−1^)	Δ*kstD*Δ*hsd4A*Δ*fadA5*		Δ*kstD*Δ*hsd4A*Δ*fadA5*-NK	
	Maximum yield (g L^−1^)	Maximum molar yield %)	Maximum yield (g L^−1^)	Maximum molar yield (%)
1	0.66 ± 0.02	78.9 ± 2.4	0.68 ± 0.04	81.2 ± 4.8
2	1.43 ± 0.06	84.8 ± 3.6	1.53 ± 0.06	90.8 ± 3.6
5	2.78 ± 0.11	66.4 ± 2.6	3.58 ± 0.15	85.5 ± 3.6
8	1.98 ± 0.08	31.5 ± 1.2	2.51 ± 0.14	38.5 ± 2.1
10	1.73 ± 0.09	20.7 ± 1.1	2.73 ± 0.19	32.6 ± 1.1

### Intracellular environmental balance contributes to higher 9-OH-4-HP production

The 9-OH-4-HP-producing strain Δ*kstD*Δ*hsd4A*Δ*fadA5* did not perform well when fed with high concentration phytosterols. This might be due to multiple factors that influence the bioconversion of phytosterols.

A series of redox reactions occur by oxygen as an electron acceptor, during phytosterols degradation, cholesterol dehydrogenases/isomerases require intracellular nicotinamide adenine dinucleotides (NAD^+^ and NADH) as cofactors [17, 18]. NAD^+^ and NADH play crucial roles during phytosterols transformation. They act in many oxidation–reduction reactions and regulate various enzymatic activities and genetic processes. One molecule of NAD^+^ accepts an H^+^ and two electrons then generates one molecule of NADH with oxidation occurrence, while the regeneration of NAD^+^ from NADH is insufficient during phytosterols side-chain degradation. Therefore, NAD^+^ and NADH have critical effects on the maintenance of the intracellular redox balance. Regeneration of NAD^+^ and enhancement of the NAD^+^/NADH ratio may be a great assistance for phytosterol transformation.

In addition, hydrogen peroxide (H_2_O_2_) is produced with incomplete oxidations during aerobic metabolism and the regeneration of flavin adenine dinucleotide (FAD) in the phytosterol transformation process [10]. A high level of H_2_O_2_ can damage proteins, DNA, and lipids in cells, resulting in an inhibition of cell growth and a low metabolite yield [19].

To enhance the ability of strain Δ*kstD*Δ*hsd4A*Δ*fadA5* to transform phytosterols into 9-OH-4-HP, the catalase KATE from *M. neoaurum* DSM 44,074 and the NADH oxidase NOX from *Bacillus subtilis* [10], were co-expressed in strain Δ*kstD*Δ*hsd4A*Δ*fadA5*, resulting in the strain Δ*kstD*Δ*hsd4A*Δ*fadA5*-NK.

**Fig. 5:**
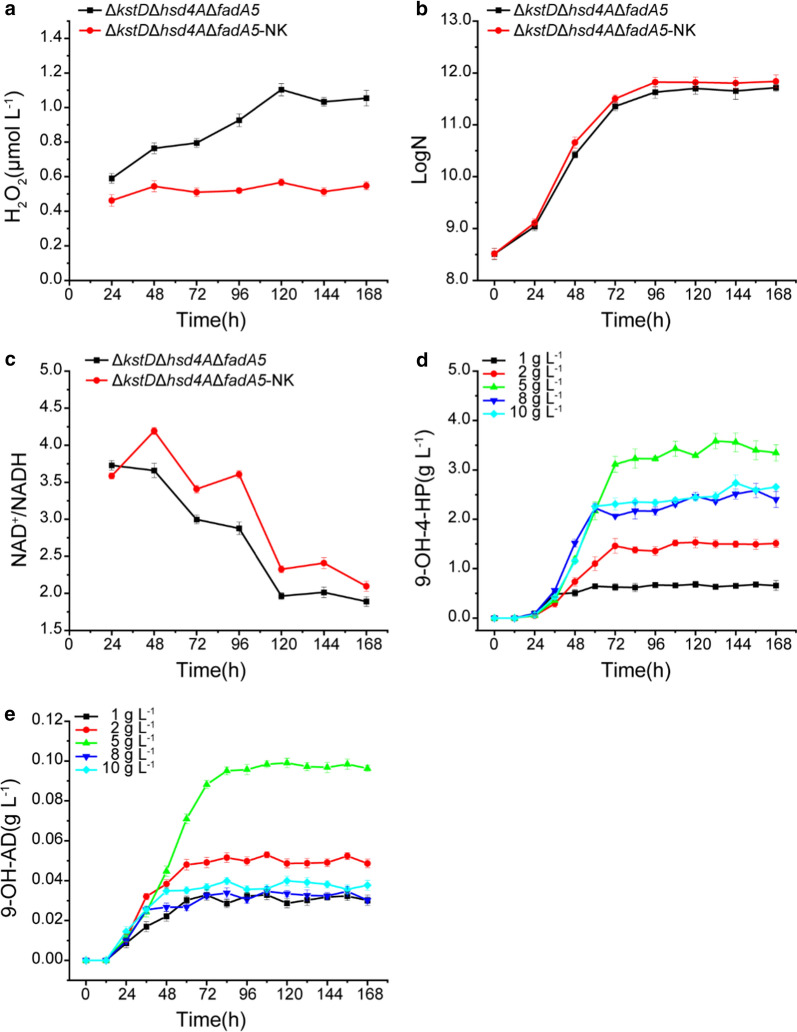
Time profiles of 9-hydroxy steroids accumulation, extracellular H_2_O_2_ concentration, and the NAD^+^/NADH ratio of the strain Δ*kstD*Δ*hsd4*AΔ*fadA5* and the strain Δ*kstD*Δ*hsd4A*Δ*fadA5*-NK. **a** Extracellular H_2_O_2_ concentration; **b** the cell growth; **c** intracellular NAD^+^/NADH ratio; **d** time course of 9-OH-4-HP accumulation of Δ*kstD*Δ*hsd4A*Δ*fadA5*-NK from different concentrations of phytosterols; **e** time course of 9-OH-AD accumulation of Δ*kstD*Δ*hsd4A*Δ*fadA5*-NK from different concentrations of phytosterols. Expression of Nox and KatE is beneficial for cell growth, increases the ratio of intracellular NAD^+^/NADH, decreases the extracellular H_2_O_2_ concentration, and enhances the yield of 9-OH-4-HP under a high concentration of phytosterol

The extracellular H_2_O_2_ concentrations of the two strains Δ*kstD*Δ*hsd4A*Δ*fadA5* and Δ*kstD*Δ*hsd4A*Δ*fadA5*-NK were measured when they were cultured with 5 g L^−1^ phytosterols for 168 h. As shown in Fig. 5a, the extracellular H_2_O_2_ concentration of strain Δ*kstD*Δ*hsd4A*Δ*fadA5* showed an upward trend during the bioconversion process. The extracellular H_2_O_2_ concentration increased from an initial concentration of 0.59 μmol L^−1^ to a final concentration of 1.05 μmol L^−1^ after 168 h. The highest concentration of H_2_O_2_ was 1.10 μmol L^−1^ at 120 h. In contrast, the extracellular H_2_O_2_ concentration of strain Δ*kstD*Δ*hsd4A*Δ*fadA5*-NK remained low and stable during the bioconversion process at approximately 0.51 μmol L^−1^, which could convince that the overexpression of *katE* eliminated excessive extracellular H_2_O_2_. Moreover, to verify the toxicity of H_2_O_2_, cell growth of the strains Δ*kstD*Δ*hsd4A*Δ*fadA5* and Δ*kstD*Δ*hsd4A*Δ*fadA5*-NK were also measured. As shown in Fig. 5b, the biomass of strain Δ*kstD*Δ*hsd4A*Δ*fadA5*-NK was higher than that of strain Δ*kstD*Δ*hsd4A*Δ*fadA5*, indicating that the elimination of extracellular H_2_O_2_ could help with cell growth.

Likewise, the NAD^+^/NADH ratios of the strains Δ*kstD*Δ*hsd4A*Δ*fadA* and Δ*kstD*Δ*hsd4A*Δ*fadA*-NK were also measured after they were cultured with 5 g L^−1^ phytosterols for 168 h. As shown in Fig. 5c, the NAD^+^/NADH ratio of strain Δ*kstD*Δ*hsd4A*Δ*fadA*-NK was consistently higher than that of strain Δ*kstD*Δ*hsd4A*Δ*fadA*. At 96 h, the NAD^+^/NADH ratio of strain Δ*kstD*Δ*hsd4A*Δ*fadA*-NK was enhanced by 25.4%, compared with that of strain Δ*kstD*Δ*hsd4A*Δ*fadA*. The overexpression of *nox* could significantly influence the NAD^+^/NADH ratio during phytosterol bioconversion.

9-OH-4-HP productivity was also measured to test whether overexpression of *katE* and *nox* could enhance the ability of Δ*kstD*Δ*hsd4A*Δ*fadA* to transform phytosterols into 9-OH-4-HP. The recombinant strain Δ*kstD*Δ*hsd4A*Δ*fadA*-NK was cultured with 1 g L^−1^, 2 g L^−1^, 5 g L^−1^, 8 g L^−1^, and 10 g L^−1^ phytosterols for 168 h, and the production of 9-OH-4-HP was measured by every 12 h. As shown in Fig. 5d, the final productions of 9-OH-4-HP from 1 g L^−1^, 2 g L^−1^, 5 g L^−1^, 8 g L^−1^, and 10 g L^−1^ phytosterols were 0.68 g L^−1^, 1.53 g L^−1^, 3.58 g L^−1^, 2.51 g L^−1^, and 2.73 g L^−1^, respectively. Compared with Δ*kstD*Δ*hsd4A*Δ*fadA*, the productions of 9-OH-4-HP were enhanced by 3.03%, 6.99%, 28.7%, 26.8%, and 57.8% under the same conditions. The highest yield of 9-OH-4-HP was obtained when strain Δ*kstD*Δ*hsd4A*Δ*fadA*-NK was cultured with 5 g L^−1^ phytosterols, with a molar yield of 85.5%,28.8% higher than that of Δ*kstD*Δ*hsd4A*Δ*fadA*. Moreover, no significant difference in the production of 9-OH-AD was observed between Δ*kstD*Δ*hsd4A*Δ*fadA5* and Δ*kstD*Δ*hsd4A*Δ*fadA5*-NK (Fig. 5e), indicating that the purity of 9-OH-4-HP was also enhanced during phytosterol bioconversion by the strain Δ*kstD*Δ*hsd4A*Δ*fadA5*-NK. All of the results above confirm controlling the intracellular NAD^+^/NADH ratio and H_2_O_2_ levels are effective to improve sterol transformation efficiency and the production of steroid intermediates.

## Discussion

By genome sequencing, three *kstDs* were found in *M. neoaurum* DSM 44074. *kstD2* and *kstD3* in *M. neoaurum* DSM 44074 showed 100% similarity with those in *M. neoaurum* ATCC 25795, a strain that was deemed to be the same strain as *M. neoaurum* DSM 44074. However, *kstD1* in *M. neoaurum* DSM 44074 showed 5 mismatches with that in *M. neoaurum* ATCC 25795, causing 3 amino acid changes. When the *kstDs* knockout strain of *M. neoaurum* DSM 44074 was cultured with phytosterols, AD and 4-HP were nearly undetectable in the final products. The *kstD* knockout strain accumulated many 9-OH-AD as the main product and little 9-OH-4-HP as a by-product. Compared with other 9-OH-AD-producing strains, the purity and molar yield of 9-OH-AD from Δ*kstD* after culture with phytosterols were notably higher. For example, *Mycobacterium* sp. 2-4 M [4] showed a 50% molar yield of 9-OH-AD, a 22% molar yield of AD, and a 2% molar yield of 4-HP from 5 g L^−1^ sitosterol [20]. In a *kstDs* knockout strain of *M. neoaurum* ATCC 25795, both the 55% molar yield of 9-OH-AD, and the 15% molar yield of AD were obtained from 15 g L^−1^ phytosterols. The accumulation of AD from *kstDs* knockout strains might be due to residual Δ^1^-dehydrogenation activity. The genome of *R. ruber* contains at least two other possible ORFs other than *kstD1*, *kstD2*, and *kstD3* with certain identities to *kstDs* (approximately 38%) [21]. The existence of more than 3 *kstDs* has also been reported for other *Rhodococcus* species, such as *R. jostii* Rha1 [22]. Thus, inactivation of all KstD may provide the fundamental premise to develop promising 9α-hydroxy derivatives producing strains.

Theoretically, lipid transfer protein Ltps catalyzes the transformation from 22-OH-24-CDOE-CoA to 4-HP (Fig. 1), but so far, no specific Ltps have been identified. Thus, manipulation of Hsd4A is usually chosen to control the metabolic flux to generate C19 steroids or C22 steroids. Xu reported the characterization of Hsd4A in vivo and in vitro, testifying that deletion of *hsd4A* resulted in blockage of the C19 steroid pathway and enhanced the accumulation of 4-HP homologues. During the Hsd4A investigation, Xu constructed a 9-OH-4-HP-producing strain by knocking out *hsd4A* in the KstD-deficient strain from *M. neoaurum* ATCC 25,795. This mutant strain displayed a 32% molar yield of 9-OH-4-HP and a 15% molar yield of 9-OH-AD from 40 g L^−1^ phytosterols [3]. Here, in this research, it was confirmed that double knockout of *hsd4A* and *fadA5* could further block the C19 steroid pathway. The molar yield of 9-OH-4-HP of strain Δ*kstDs*Δ*hsd4A*Δ*fadA5* was notably higher than those of Xu’s strain, which is 90.8% versus 32%.

As shown in Table 2, the molar yield of 9-OH-4-HP decreased with the increase of phytosterols concentration. This might be due to the hindrance of phytosterols. On one hand, the aqueous solubility of phytosterols is too low to form a dispersive state [23], which impedes the contact within cells and further retard the phytosterols bioconversion. Therefore, with the phytosterol concentration increasing, phytosterol is hard to form dispersive solution, and further resulting in low molar yields of sterol derivatives productions. The major limitation on microbial transformation of phytosterols is due to its low solubility in aqueous media. The conversion yields can be improved by adding surface active agents to the transformation media, such as Tween 80 or Triton X-100, and other sterol-solubilizing agents like cyclodextrins [24]. Some surfactants like Tween 80 can reduce the tendency of *Mycobacterium* to aggregate [25] and thus promote the formation of stable suspensions of phytosterol by increasing phytosterol solubility and decreasing dynamic interfacial tension [23].These above cause the improvement of phytosterol bioconversion. Sterol-solubilizing agents like cyclodextrins could form inclusion complexes with steroids and make them as the effective carriers to deliver of hydrophobic steroids to cells, which enhances the biotransformation of steroid compounds [26]. Phytosterols and their derivatives are also reported to be toxic and inhibitory to cell growth [26, 27], which also hinder the bioconversion and result in low molar yields. Balancing the intracellular environment is another promising method to improve phytosterols bioconversion. On one hand, NAD^+^ is greatly consumed during phytosterol bioconversion, which participates in many reactions of phytosterol side-chain degradation. On the other hand, phytosterols’ toxicity to cells impedes the bioconversion. In this study, when combining the abilities of NAD^+^ regeneration and H_2_O_2_ elimination, the performance of the new mutant strain Δ*kstDs*Δ*hsd4A*Δ*fadA5*-NK to transform phytosterols into 9-OH-4-HP was improved. The extracellular H_2_O_2_ concentration of Δ*kstDs*Δ*hsd4A*Δ*fadA5*-NK, after culture with 5 g L^−1^ phytosterols, decreased 49.4% due to the overexpression of *katE*. The NAD^+^/NADH ratio was also enhanced by 25.4% after 96 h with the overexpression of *nox*. Although the molar yield of 9-OH-4-HP decreased with increasing of the phytosterol concentration, the molar yield of 9-OH-4-HP from strain Δ*kstDs*Δ*hsd4A*Δ*fadA5*-NK was significantly higher than that from strain Δ*kstDs*Δ*hsd4A*Δ*fadA5* at the same concentration phytosterols. The highest yield of 9-OH-4-HP was 3.58 g L^−1^ from Δ*kstDs*Δ*hsd4A*Δ*fadA5*-NK with 5 g L^−1^ phytosterols fed, which is 28.7% higher than that from strain Δ*kstDs*Δ*hsd4A*Δ*fadA5*. Again, these results proved that the elimination of H_2_O_2_ and regeneration of NAD^+^ are effective methods to improve phytosterol bioconversion.

The accumulation of C19 steroids in the Hsd4A and/or FadA5 deficiency strains indicates incomplete blockage of the C19 steroid pathway. Similar results have been previously reported [3, 28]. An Hsd4A2 was testified to be dominant in *M. neoaurum* CCTCC AB2019054 rather than Hsd4A [28]. A protein in *M. neoaurum* DSM 44074 of 98.67% identity with Hsd4A2 in *M. neoaurum* CCTCC AB2019054 was found by blastp, indicating Hsd4A2 existing in *M. neoaurum* DSM 44074. Analysis of the *M. neoaurum* DSM 44074 genome implied more potential Hsd4A isoenzymes. Thus, the downstream gene of *hsd4A*, *fadA5*, was chosen to be knocked out to reduce the accumulation of 9-OH-AD ulteriorly. However, 9-OH-AD still took up 2% of the products when Hsd4A and FadA5 deficiency strain was cultured with phytosterols. Similarly, analysis of the *M. neoaurum* DSM 44074 genome implied FadA5 isoenzymes existing, which could also explain why the strain with FadA5 deficiency also accumulated 9-OH-AD.

## Conclusion

This study aimed to construct an efficient 9-OH-4-HP production strain, which is a novel and valuable steroid drugs precursor. The higher accumulation of 9-OH-4-HP was achieved by *hsd4A* and *fadA5* knockout to block the C19 steroid pathway and by 3-ketosteroid-Δ^1^-dehydrogenation deficiency with *kstDs* knockout. Compared with the Hsd4A deficiency or the FadA5 deficiency, the double deletion of *hsd4A* and *fadA5* could further block the C19 steroid pathway in phytosterol side-chain degradation. By eliminating H_2_O_2_ and regenerating NAD^+^ in the KstD, Hsd4A, and FadA5 deficiency strain, the 9-OH-4-HP yield was significantly improved. These findings provided some new insights into the accumulation of C22 steroids and methods by improving phytosterols bioconversion.

## Methods

### Bacterial strains, plasmids, medium, and reagents

The stains and plasmids used in this study are described in Table 3. *E. coli* DH5α stored in the laboratory was used for plasmid amplification. Wild-type *M. neoaurum* DSM 44074 (DSM 44704) was purchased from Deutsche Sammlung von Mikroorganismenund Zellkulturen (DSMZ, GERMANY). All other strains were derived from *M. neoaurum* DSM 44704. Common plasmids and primers (Additional file 1: Table S1) were used to construct the mutants. *E. coli* DH5α was cultured at 37 °C and 200 rpm in 50 mL of Luria–Bertani (LB) medium (10 g L^−1^ tryptone, 10 g L^−1^ NaCl, 5 g L^−1^ yeast extracts, pH 7.0). *Mycobacterium* cells were cultured in MYD medium (0.6 g L^−1^ K_2_HPO_4_·3H_2_O, 5.4 g L^−1^ NaNO_3_, 6 g L^−1^ glucose, 15 g L^−1^ yeast extract and an initial pH value 7.5) and fermented with MP01 medium (10 g L^−1^ corn steep powder, 20 g L^−1^ glucose, 2 g L^−1^ K_2_HPO_4_·3H_2_O, 1.0 g L^−1^ MgSO_4_·7H_2_O, 2.0 g L^−1^ NaNO_3_, 2‰ Tween 80 (v/v), and an initial pH value 7.5) at 30 °C and 200 rpm.

**Table 3:** Strains and plasmids used in this study

Name	Description	Source
Strains		
*M. neoaurum* DSM 44704	Wild type strain, Sterol consumer with no detectable intermediates	
Δ*kstD*	*kstD1*, *kstD2*, and *kstD3* deleted in *M. neoaurum* DSM 44704	This study
Δ*kstD*Δ*hsd4A*	*hsd4A* deleted in Δ*kstD* strain	This study
Δ*kstD*Δ*fadA5*	*fadA5* deleted in Δ*KstD* strain	This study
Δ*kstD*Δ*hsd4A*-*hsd4A*	Δ*kstD*Δ*hsd4A* strain harboring P38Mu-Hsd4A	This study
Δ*kstD*Δ*fadA5*-*fadA5*	Δ*kstD*Δ*fadA5* strain harboring P38Mu-FadA5	This study
Δ*kstD*Δ*hsd4A*Δ*fadA5*	*hsd4A* and *fadA5* deleted in *ΔkstD* strain	This study
Δ*kstD*Δ*hsd4A*Δ*fadA5*-NK	Δ*kstD*Δ*hsd4A*Δ*fadA5* strain harboring P38Mu-NK	This study
Plasmids		
PSBY1	Derived from pMV261 and contains FnCpf1 *C*. *glutamicum* codon-optimized; KanR	[29]
PCR-Hyg	Plasmid for sgRNA production	[30]
Pam-KstD1	PCR-Hyg containing *kstD1* spacer	This study
Pam-KstD2	PCR-Hyg containing *kstD2* spacer	This study
Pam-KstD3	PCR-Hyg containing *kstD3* spacer	This study
Pam-Hsd4A	PCR-Hyg containing *hsd4A* spacer	This study
Pam-FadA5	PCR-Hyg containing *fadA5* spacer	This study
pMV306	*Mycobacterium* integrative vector without promoter, kan^R^	[31]
P38Mu	pMV306 with Psmyc promoter, Kan^R^	[32]
P38Mu-Hsd4A	Recombinant P38Mu for expression of *hsd4A*	This study
P38Mu-FadA5	Recombinant P38Mu for expression of *fadA5*	This study
P38Mu-NK	Recombinant P38Mu for expression of *nox* and *katE*	This study

The phytosterols consisted of 45% β-sitosterol, 37% campesterol, and 18% stigmasterol, which were purchased from Yunnan Biological Products Co., Ltd. (Yunnan, China). AD and 9-OH-AD were obtained from Shanghai Macklin Biochemical Co., Ltd. (China). (2-Hydroxypropyl)-β-cyclodextrin (HP-β-CD) was purchased from Zhiyuan Biotechnology Co., Ltd. (Shandong, China). The ClonExpressIIOne Step Cloning Kit was purchased from Vazyme Biotech Co., Ltd. (Nanjing, China). The Hydrogen Peroxide (H_2_O_2_) Content Assay Kit and Nicotinamide Adenine Dinucleotide NAD(H) Content Assay Kit were obtained from Sangon Biotech Co., Ltd. (Shanghai, China).

### Bioinformatic analysis

The genome of *M. neoaurum* DSM 44,074 was sequenced by Shanghai Majorbio Co., Ltd. The DNA sample was extracted and sheared into 400–500 bp fragments using a Covaris M220 Focused Acoustic Shearer (Covaris, USA). Illumina sequencing libraries were prepared from the sheared fragments using a NEXTflex™ Rapid DNS-Seq Kit (Bioo Scientific, USA). The sequencing data were assembled using SOAPdenovo2(GitHub—aquaskyline/SOAPdenovo2: Next generation sequencing reads de novo assembler.). Further prediction and annotation were produced by Glimmer (Glimmer (jhu.edu)) and BLAST (blast.ncbi.nlm.nih.gov). The putative genes for *kstD*, *hsd4A*, *and fadA5* were identified by comparison with known gene sequences taken from the NCBI database. MEGA-X software (Home (megasoftware.net)) was used to construct a phylogenetic tree of *hsd4A* and *fadA5* with the known amino acid sequences taken from the NCBI.

### Mutant strain construction

A CRISPR-assisted nonhomologous end-joining strategy was used to delete the target gene in *M. neoaurum* DSM 44,074 based on previous reports. The PSBY1 plasmid harbouring cpf1 was obtained from Jiang [29], and the PCR-Hyg plasmid harbouring sgRNA was obtained from Sun [30]. ClonExpressIIOne Step Cloning Kit mutated spacers were used to construct different plasmids harbouring target sgRNA. The plasmid harbouring target sgRNA was transfected into *M. neoaurum*, and the PSBY1 plasmid was transfected beforehand by electroporation. The recombinant clones were sequenced using specific primers to determine the deletion.

The vector P38Mu (pMV306 with the Psmyc promoter) with kanamycin resistance was used to overexpress the target gene. The genes *hsd4A*, *fadA5, katE* from *M. neoaurum* DSM 44074, and *nox* from *Bacillus subtilis* were recombined on P38Mu. Specific primers were used to amplify the corresponding gene, and the PCR product was inserted into the NdeI site (and HindIII site, if two genes were inserted) of P38Mu using the ClonExpressIIOne Step Cloning Kit.

### Bioconversion and analysis

The transformation capability of the mutant strains was identified in MP01 medium with an initial phytosterol concentration of 1 g L^−1^. A concentration gradient was later tested to further determine the capability of phytosterol bioconversion. Phytosterols were prepared in (2-hydroxypropyl)-β-cyclodextrin (HP-β-CD) at a ratio of 1:1.5. The recombinant cells were inoculated into 30 mL of MYD medium in a 250 mL shaker flask and cultured at 30 °C and 200 rpm. Three mL of seed medium was transferred to 30 mL of MP01 medium in a 250 mL shaker flask with a baffle when the optical density reached the mid-log exponential phase. The fermentation of *M. neoaurum* DSM 44,074 and recombinant strains was sampled every 12 or 24 h, and three replicates were used to measure the steroids. The bioconversion mixture was extracted with 3 volumes of ethyl acetate, and the solvent was removed to give a residue that was redissolved in methanol. The resulting solution was used for HPLC analysis. HPLC was performed on a Shimadzu Separations module connected to a Shimadzu SPD-M20A detector equipped with a C18 column (250 mm × 4.6 mm, 5 µm) and detected at a wavelength of 254 nm. A mixture of methanol and water (80:20, v/v) was used as the mobile phase at a flow rate of 0.8 mL min^−1^.

Extracellular H_2_O_2_ concentrations were measured according to the operating manual of the Hydrogen Peroxide (H_2_O_2_) Content Assay Kit. NADH and NAD^+^ intracellular concentrations were measured according to the operating manual of the Nicotinamide Adenine Dinucleotide, NAD(H) Content Assay Kit.

## Supplementary Information

**Additional file 1: Table S1.** Primers used in this work. **Fig. S1.** Cell growth of *M. neoaurum* DSM 44074 and its mutant strains. **Fig. S2.** Phylogenetic trees of Hsd4A and FadA5.

## Data Availability

All data generated and analyzed during this study are included in this published article and its additional files.

## Abbreviations

AD: Androst-4-ene-3,17-dione
ADD: Androst-1,4-diene-3,17-dione
9-OH-AD: 9-Hydroxy-androst-4-ene-3,17-dione
4-HP: 21-Hydroxy-20-methyl-pregna-4-en-3-one
1,4-HP: 21-Hydroxy-20-methyl-pregna-1,4-dien-3-one
9-OH-4-HP: 9,21-Dihydroxy-20-methyl-pregna-4-en-3-one
KstDs: 3-Ketosteroid-Δ^1^-dehydrogenases
KSHs: 9α-Hydroxylase
HPs: C22 steroids
Hsd4A: β-Hydroxyacyl-CoA dehydrogenase
FadA5: Acyl-CoA thiolase
ROS: Reactive oxygen species
H_2_O_2_: Hydrogen peroxide
NAD^+^/NADH: Nicotinamide adenine dinucleotides
NOX: NADH oxidase
KatE: Catalase
HPLC: High performance liquid chromatography
