# Supplementary material for: Production of 9,21-dihydroxy-20-methyl-pregna-4-en-3-one from phytosterols in Mycobacterium neoaurum by modifying multiple genes and improving the intracellular environment
Source: Microb Cell Fact. 2021 Dec 23;20:229. doi: 10.1186/s12934-021-01717-w (PMC8705162; doi:10.1186/s12934-021-01717-w)

Table S1 Primers used in this study

| Name | Nucleotide |
| --- | --- |
| K1-F | GTTCTACATGACTGAACAGG |
| K1-R | TCAGGCCTTTCCAGCGAGAT |
| K2-F | GTGACCGACCAGAAGAACAT |
| K2-R | GGCGTGGTGAGCCGCGATAT |
| K3-F | AGTTCGATGTCATCGTCGCC |
| K3-R | TCATTCCGCTGAGCTCTGTG |
| Hsd4A-F | CGGCCCTGATCCGTAGAGAC |
| Hsd4A-R | GCCGGTGTGACGGATGTCAT |
| FadA5-F | TGGGTAATCCTGTCATCGTC |
| FadA5-R | TAGATCCGCTCGATGATGGT |
| Pam-K1-F | ATCTCGCCGAGGTTCGGGTTCGGCTGTCTAAGAACTTTAAATAATTTCTACTGTTGTAGATATCGACTGCCAGGCATCAAA |
| Pam-K1-R | AGCCGAACCCGAACCTCGGCGAGATATCTACAACAGTAGAAATTATTTAAAGTTCTTAGACCCGTTTTTGCCTAAATCAGC |
| Pam-K2-F | GCTGATTTAGGCAAAAACGGGTCTAAGAACTTTAAATAATTTCTACTGTTGTAGATCACGATCAACGTCGAGAGCCCCAGT |
| Pam-K2-R | TTTGATGCCTGGCAGTCGATATCTACAACAGTAGAAATTATTTAAAGTTCTTAGACACTGGGGCTCTCGACGTTGATCGTG |
| Pam-K3-F | ACGGGGGGAATGTCGCCGTCGCGGTGTCTAAGAACTTTAAATAATTTCTACTGTTGTAGATATCGACTGCCAGGCATCAAA |
| Pam-K3-R | ACCGCGACGGCGACATTCCCCCCGTATCTACAACAGTAGAAATTATTTAAAGTTCTTAGACCCGTTTTTGCCTAAATCAGC |
| Pam-Hsd4A-F | CGGACAGGTCGATCGGGTTGTCGTTGTCTAAGAACTTTAAATAATTTCTACTGTTGTAGATATCGACTGCCAGGCATCAAA |
| Pam-Hsd4A-R | AACGACAACCCGATCGACCTGTCCGATCTACAACAGTAGAAATTATTTAAAGTTCTTAGACCCGTTTTTGCCTAAATCAGC |
| Pam-FadA5-F | GCGTCCGTCGTGCTGTCCTGGGCGCGTCTAAGAACTTTAAATAATTTCTACTGTTGTAGATATCGACTGCCAGGCATCAAA |
| Pam-FadA5-R | GCTGATTTAGGCAAAAACGGGTCTAAGAACTTTAAATAATTTCTACTGTTGTAGATGCGTCCGTCGTGCTGTCCTGGGCGC |
| P38Mu-Hsd4A-F | TAAGAAGGAGATATACATATGAACGACAACCCGATCGACC |
| P38Mu-Hsd4A-R | GATGAATTCGGATCCTCAAGAGCCCATGAGCTCGG |
| P38Mu-FadA5-F | TAAGAAGGAGATATACATATGGGTAATCCTGTCATCGTCG |
| P38Mu-FadA5-R | GATGAATTCGGATCCTTAGATCCGCTCGATGATGGTG |
| P38Mu-F | TTCCTGGCCTTTTGCTGGCC |
| P38Mu-R | GCGTTCGCCCTGTCGTTCAC |
| P38Mu-NOX-F | ATACATATGGGATCCGAATTCATGACGAATACTCTGGATGTTTTAAAA |
| P38Mu-NOX-R | CCGAAGCTTATCGATTTACAGCCAAGTTGATACTTTTGAAA |
| P38Mu-KatE-F | TGGCTGTAAATCGATAAGCTTAAGAAGGAGATATAATGCGCGAGAGGAACACC |
| P38MU-Kate-R | GAAGTGATTCCTCCGCTACTTGACTGCCGCCTCGA |

Figure S1. Cell growth of *M.neoaurum* DSM 44074 and its mutant strains


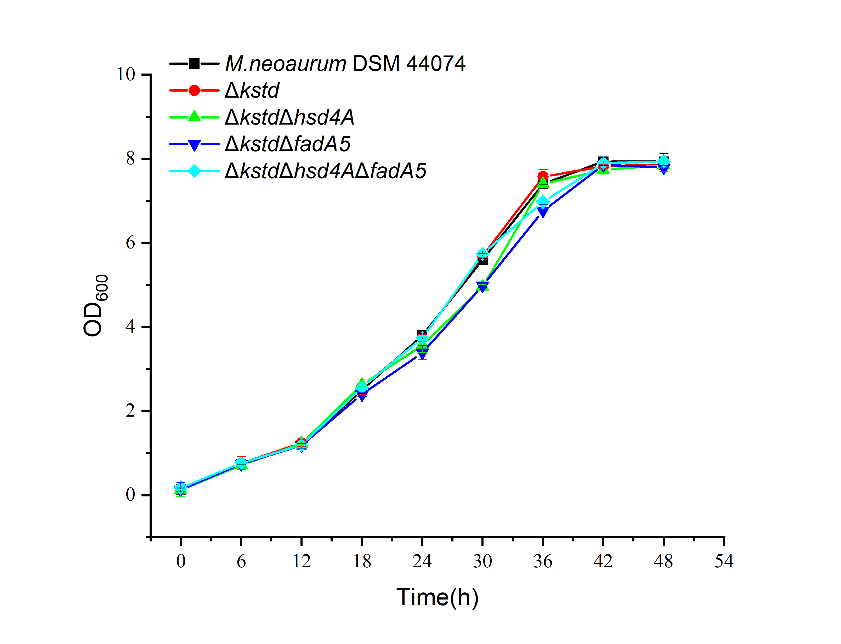


Figure S2. Phylogenetic trees of Hsd4A and FadA5

1. Phylogenetic tree of Hsd4A; (b) Phylogenetic tree of FadA5


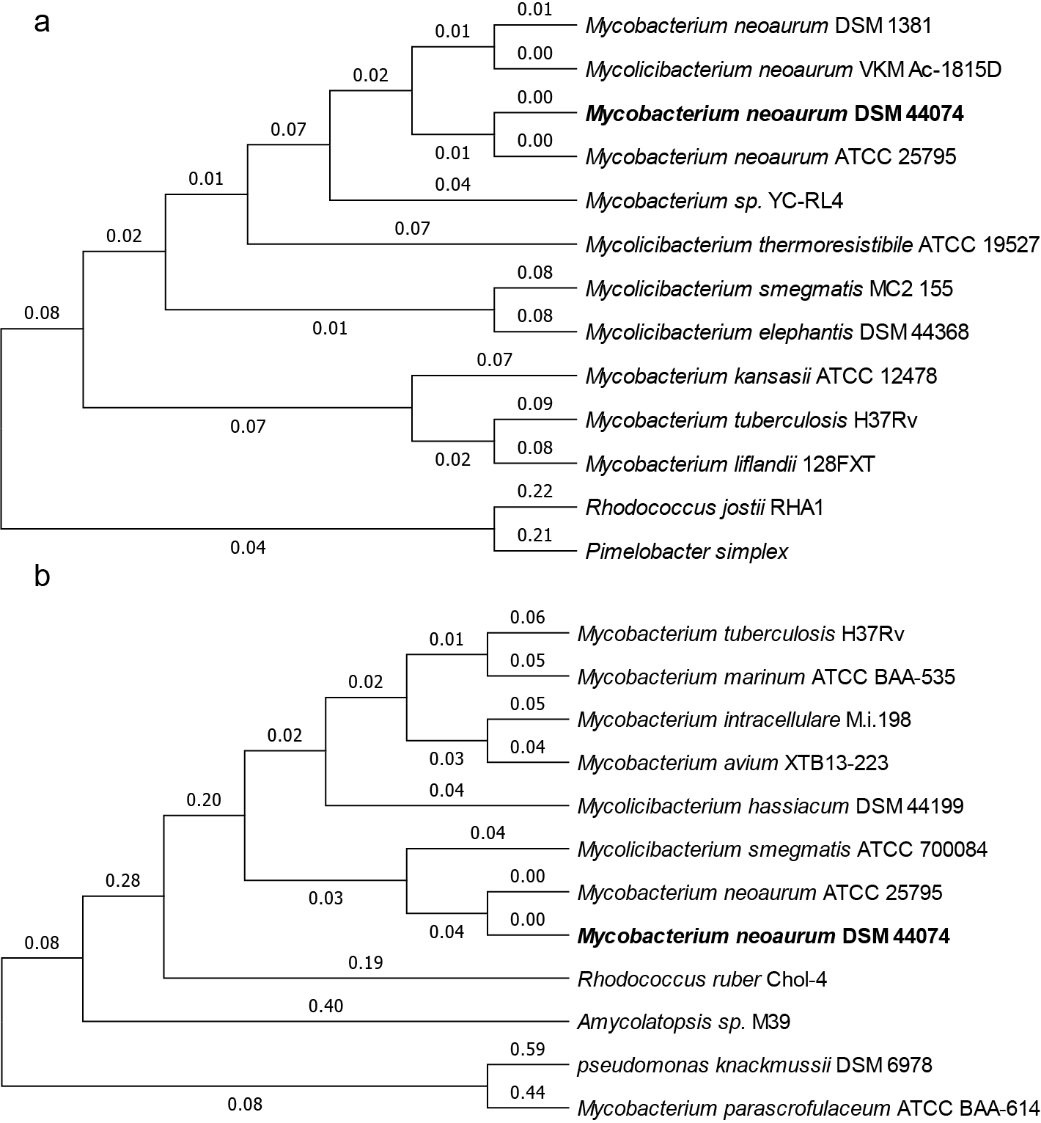

Supplement: Supplementary file 1 — Additional file 1: Table S1. Primers used in this work. Fig. S1. Cell growth of M. neoaurum DSM 44074 and its mutant strains. Fig. S2. Phylogenetic trees of Hsd4A and FadA5. [file 12934_2021_1717_MOESM1_ESM.docx]
